# A Blind Spot in Imaging: Immune Checkpoint Inhibitor-Induced Myocarditis with Negative Cardiac MRI

**DOI:** 10.14797/mdcvj.1597

**Published:** 2025-05-01

**Authors:** Lubna Alnatour, Ayham Mahmoud, Ameer Awashra, Jonathan Na, Mohammed Chamsi-Pasha

**Affiliations:** 1 School of Medicine, University of Jordan, Amman, Jordan; 2 School of Medicine, The University of Jordan, Amman, Jordan; 3 An Najah National University, Nablus, Palestine; 4 Houston Methodist, Houston, Texas, US; 5 Houston Methodist DeBakey Heart & Vascular Center, Houston, Texas, US

**Keywords:** immune checkpoint inhibitors (ICI)-induced myocarditis, myocarditis, CMR, cardio-oncology

## Abstract

Immune checkpoint inhibitor (ICI)-induced myocarditis is rare but carries a high morbidity and mortality rate. While cardiac magnetic resonance imaging (CMR) is the first-line imaging modality to support diagnosis, the need for endomyocardial biopsy is needed in negative CMR cases.

A 57-year-old male with a known case of oral cavity squamous cell carcinoma presented to the emergency department with chest pain. His current chemotherapy regimen included carboplatin, paclitaxel, and pembrolizumab (anti-programmed cell death protein 1 therapy). The patient appeared ill, with resting tachycardia (heart rate of 101 bpm) and mildly elevated jugular venous pressure at 10 cm. Labs showed elevated troponin 336 ng/L, NT-proBNP 252 pg/mL. An electrocardiogram showed sinus tachycardia and no acute ST-T-wave changes (Figure 2 A).

Transthoracic echocardiogram showed preserved ventricular function and small pericardial effusion with respiratory variation across the mitral valve consistent with increased intrapericardial pressures (Figure 1 A–C, Video 1). The hospital course was notable for ongoing myocardial injury (troponin 1185 ng/L), new right bundle branch block (Figure 1 B), and episodes of nonsustained ventricular tachycardia. To rule out other non-myocardial infarction causes of elevated troponin, cardiac magnetic resonance imaging (MRI) was performed, showing borderline reduced systolic function (left ventricle ejection fractions 51%) and moderate (10 mm) circumferential pericardial effusion but with no inflammation (Figure 1 D, F asterisks).

**Figure 1:**
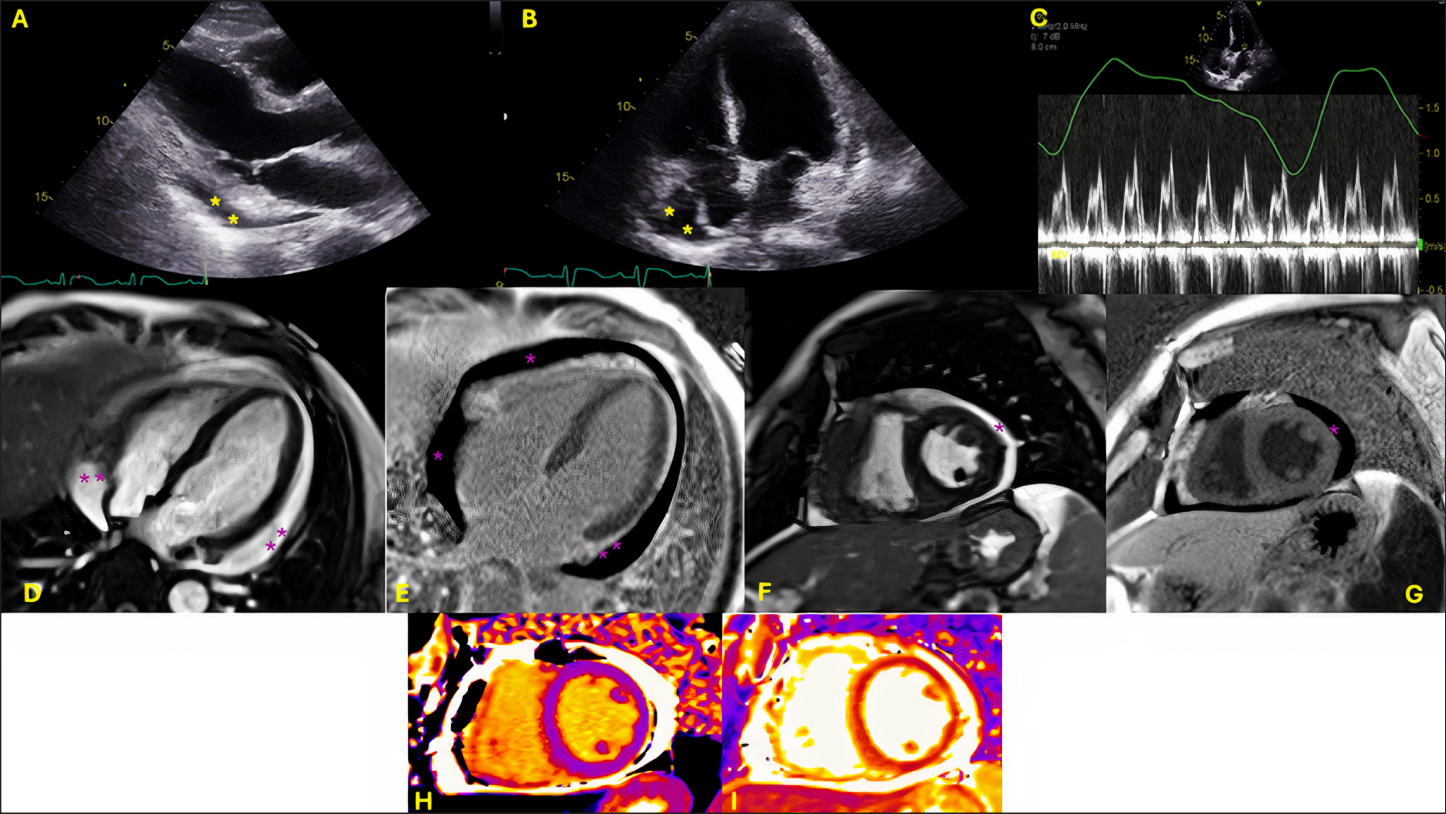
**(A)** Transthoracic echocardiogram, parasternal long-axis image with small posterior pericardial effusion (asterisk), **(B)** transthoracic echocardiogram, apical 4-chamber view with anterior pericardial effusion (asterisk). **(C)** Pulsed wave Doppler across the mitral inflow showing respiratory variation > 25% consistent with increased intrapericardial pressures. **(D, F)** Cardiovascular magnetic resonance imaging, balanced steady-state free precession sequence in apical 4-chamber **(D)** and short-axis view **(F)** showing circumferential pericardial effusion (moderate, asterisk). **(E, G)** Late gadolinium enhancement imaging suggests nulled myocardium with no evidence of ischemic damage or myocarditis. **(H, I)** T1 and T2 parametric mapping.

**Video 1:** Cine steady-state free precession cardiac MRI sequence (real-time imaging) in short axis stacks across the LV, 4-chamber view, 2-chamber view, and 3-chamber view. There is circumferential pericardial effusion but no evidence of chamber collapse. The left ventricular function is borderline reduced; also view at https://youtube.com/shorts/mFHytiYCu5k.

**Figure 2:**
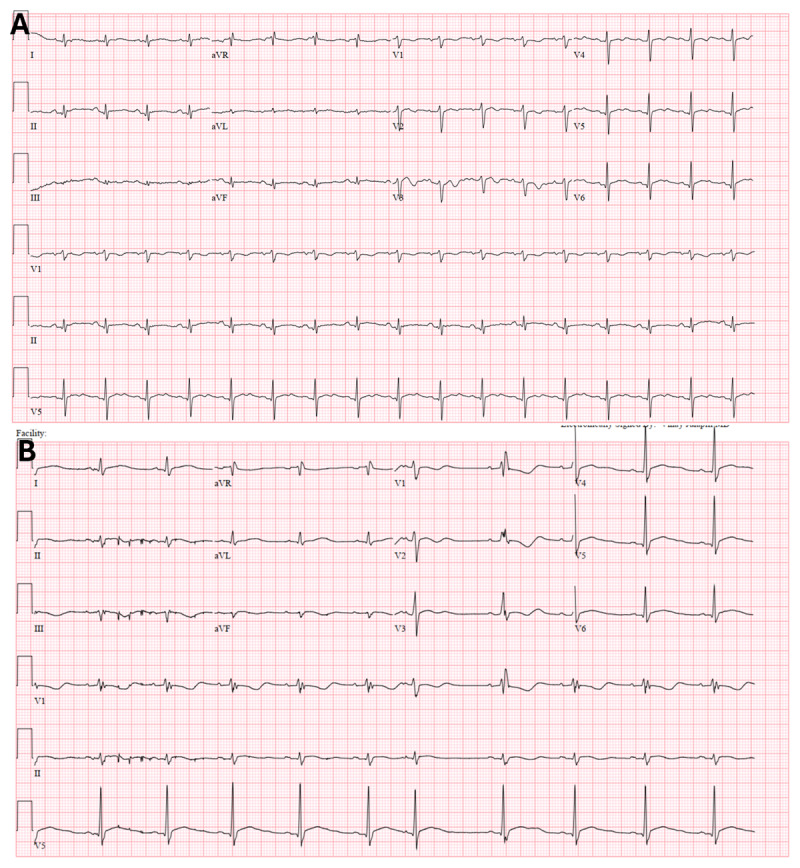
**(A)** 12-lead electrocardiography showing sinus tachycardia but no acute ST-T wave changes. **(B)** Subsequent electrocardiogram showing interval development of right bundle branch block.

There was no evidence of late gadolinium enhancement suggesting absence of infarction, acute injury, or myocarditis (Figure 1 E, G). In addition, the T1 and T2 relaxation times were normal on parametric mapping (Figure 1 H, I). Given the possibility of immune-checkpoint inhibitor (ICI)-myocarditis, the patient underwent an endomyocardial biopsy, which showed increased perivascular and interstitial CD3 and CD8 positive T cells, findings consistent with ICI- myocarditis (Figure 3). The patient completed five doses of pulsed intravenous steroids and was discharged home on 60 mg prednisone with weekly taper. Immunotherapy was permanently discontinued.

**Figure 3:**
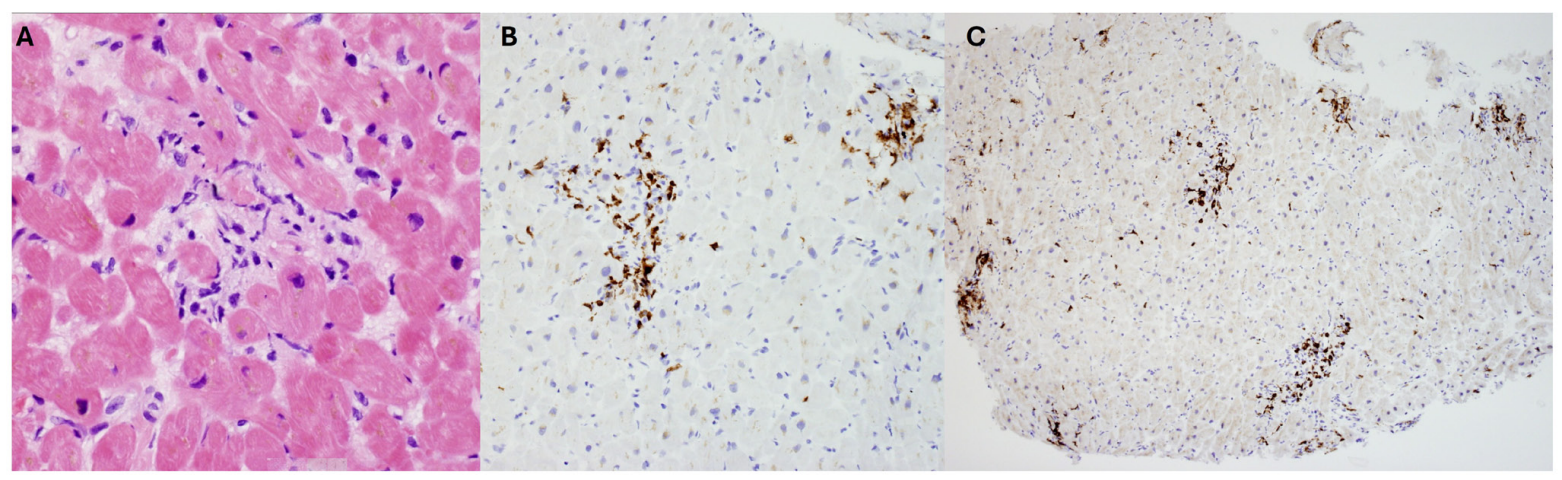
**(A)** Hematoxylin and eosin stain showing increase perivascular and interstitial CD3 positive **(B)** and CD8 **(C)** T-cells, findings compatible with immune checkpoint inhibitor-associated myocarditis.

First reported in 2016 and infrequently encountered,^1^ this case demonstrates the need for increased awareness of ICI myocarditis, which carries a 40% risk of morbidity and mortality. Early diagnosis with interruption of ICI and initiation of immunosuppression is key to improving outcomes.^2^
